# Kalirin-7 is necessary for normal NMDA receptor-dependent synaptic plasticity

**DOI:** 10.1186/1471-2202-12-126

**Published:** 2011-12-19

**Authors:** Fouad Lemtiri-Chlieh, Liangfang Zhao, Drew D Kiraly, Betty A Eipper, Richard E Mains, Eric S Levine

**Affiliations:** 1 Department of Neuroscience, University of Connecticut Health Center, Farmington, CT 06030 USA; 2 The King Abdullah University of Science and Technology, Thuwal, 23955-6900, Kingdom of Saudi Arabia

## Abstract

**Background:**

Dendritic spines represent the postsynaptic component of the vast majority of excitatory synapses present in the mammalian forebrain. The ability of spines to rapidly alter their shape, size, number and receptor content in response to stimulation is considered to be of paramount importance during the development of synaptic plasticity. Indeed, long-term potentiation (LTP), widely believed to be a cellular correlate of learning and memory, has been repeatedly shown to induce both spine enlargement and the formation of new dendritic spines. In our studies, we focus on Kalirin-7 (Kal7), a Rho GDP/GTP exchange factor (Rho-GEF) localized to the postsynaptic density that plays a crucial role in the development and maintenance of dendritic spines both *in vitro *and *in vivo*. Previous studies have shown that mice lacking Kal7 (Kal7^KO^) have decreased dendritic spine density in the hippocampus as well as focal hippocampal-dependent learning impairments.

**Results:**

We have performed a detailed electrophysiological characterization of the role of Kal7 in hippocampal synaptic plasticity. We show that loss of Kal7 results in impaired NMDA receptor-dependent LTP and long-term depression, whereas a **NMDA receptor-independent **form of LTP is shown to be normal in the absence of Kal7.

**Conclusions:**

These results indicate that Kal7 is an essential and selective modulator of NMDA receptor-dependent synaptic plasticity in the hippocampus.

## Background

Dendritic spines are the locus of the majority of excitatory synapses on hippocampal and cortical pyramidal neurons. An abundance of research in the field of synaptic plasticity has demonstrated that dendritic spines display morphological plasticity in response to a myriad of extracellular stimuli [1,2]. These changes are thought to be cellular correlates of the plasticity seen in learning and memory [3]. Importantly, spines have repeatedly been shown to increase in both size and number following the induction of long-term potentiation (LTP) [4-7] and to decrease in size and number following induction of long-term depression (LTD) [8,9]. The ability of dendritic spines to remain labile/plastic is dependent on rearrangement of the actin cytoskeleton which forms the core of each spine [10-12]. This process is dependent on the activity of Rho-GTPases, which are activated by Rho-guanine nucleotide exchange factors (Rho-GEFs) [13]. About a dozen of the 58 Rho-GEFs encoded by the mouse genome are localized to the postsynaptic density (PSD) [14].

Among the PSD-localized Rho-GEFs is Kalirin-7 (Kal7), the predominant adult splice variant of the multiply spliced *Kalrn *gene [15,16]. Kal7 has been repeatedly shown to have a profound effect on dendritic spine density *in vitro*, with over-expression dramatically increasing spine density and knockdown decreasing spine density [17,18]. More recently, we developed a mouse that cannot produce Kal7 (Kal7^KO^) and demonstrated that this mouse had decreased hippocampal spine density at baseline, and was unable to increase dendritic spine density in medium spiny neurons in the nucleus accumbens in response to repeated cocaine treatment [17,19].

Electrophysiologically, genetic deletion of Kal7 resulted in a decrease in the frequency of spontaneous excitatory postsynaptic potentials (sEPSPs) with no change in sEPSP amplitude, suggesting that expression of AMPA receptors at existing synapses was normal, while synapse number was reduced [17]. A similar decrease in sEPSP frequency was seen in cortical neurons in an animal unable to produce any of the full length Kalirin isoforms due to deletion of exons in the first GEF domain of Kalirin (KalGEF1^KO^) [20]. Interestingly, Kal7^KO ^mice exhibited a robust decrease in LTP in the hippocampus [17] whereas KalGEF1^KO ^mice demonstrated a small but significant decrease in hippocampal field LTP [21]. Recent biochemical studies revealed a direct interaction between Kal7 and the NR2B subunit of the NMDA receptor and NMDA receptor-mediated transmission was shown to be significantly impaired in the cortex of Kal7^KO ^mice [22].

In this series of experiments, we characterized basal synaptic transmission and synaptic plasticity in the hippocampus of Kal7^KO ^mice. We found that Kal7^KO ^mice exhibit normal AMPA receptor-mediated basal transmission, profound deficits in NMDA receptor-dependent LTP and LTD, and normal **NMDA receptor-independent **plasticity. These studies shed light on the specific pathways that are affected by the presence or absence of Kal7 at a synapse.

## Methods

### Ethical approval

All animal procedures were conducted according to protocols approved by the University of Connecticut Health Center Institutional Animal Care and Use Committee.

### Slice preparation

**Figure 1:**
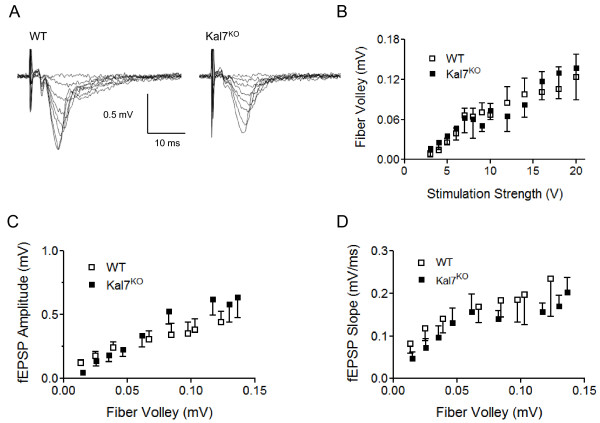
**Basal AMPA receptor-mediated synaptic transmission is not altered by Kal7 deletion**. A) Example traces of evoked field EPSPs (fEPSPs) in WT and Kal7^KO ^mice. B) Input-output curve showing the relationship between stimulation intensity and presynaptic fiber volley for evoked AMPA receptor-mediated hippocampal fEPSPs. This is a measure of the excitability of the presynaptic axons that are being stimulated (WT: n = 8 slices from 3 animals; Kal7^KO^: n = 6 slices from 3 animals). C) Input-output curve showing relationship between presynaptic fiber volley and fEPSP amplitude for the same slices as in B. D) Input-output curve showing relationship between presynaptic fiber volley and fEPSP slope for the same slices as in B. There was no significant difference between WT and Kal7^KO ^for either amplitude or slope.

**Figure 2:**
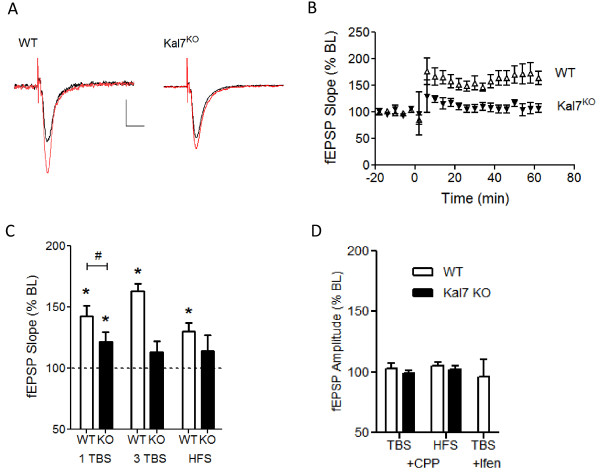
**NMDA receptor-dependent LTP is impaired in Kal7^KO ^animals**. A) Example traces of evoked fEPSPs before (black) and 60 min after (red) 3 trains of theta burst stimulation (TBS) for a WT and Kal7^KO ^animal. Each TBS train consisted of 10 bursts delivered at 5 Hz, and each burst consisted of 5 stimuli at 100 Hz. Calibration bars: 0.1 mV, 20 ms. Example traces in this and subsequent figures are averages of 10-15 sweeps. B) Group time courses of fEPSP slope recorded from WT (n = 5 animals) and Kal7^KO ^mice (n = 5) following 3 trains of TBS delivered at time zero. C) Group data showing magnitude of LTP measured 60 min post-induction for 1 train of TBS (WT: n = 8; Kal7^KO^: n = 7), 3 trains of TBS (n = 5 for both WT and Kal7^KO^), or high frequency stimulation (HFS; 100 Hz/1 sec; n = 5 for both WT and Kal7^KO^). *, p < 0.05 compared to baseline; #, p < 0.05 between condition. D) LTP induced by TBS or HFS requires NR2B-containing NMDA receptors. Group data showing the lack of TBS-induced or HFS-induced LTP in the presence of the NMDA receptor antagonist CPP (3 μM) in both WT and Kal7^KO ^animals (n = 3-5 slices/condition), and the lack of TBS-induced LTP in WT animals in the presence of the NR2B antagonist ifenprodil (3 μM; n = 5).

**Figure 3:**
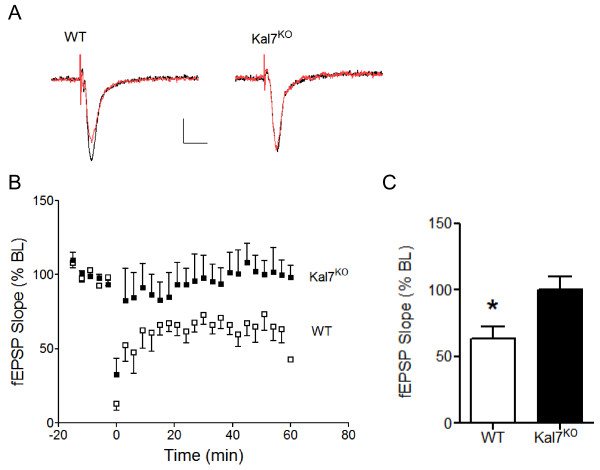
**Long-term depression (LTD) is disrupted in Kal7^KO ^mice**. A) Example traces of evoked fEPSPs before (black) and 30 min after (red) paired-pulse low frequency stimulation (PP-LFS; 3 stimuli/50 ms intervals delivered at 1 Hz for 15 min) for a WT and Kal7^KO ^animal. Calibration bars: 0.1 mV, 20 ms. B) Group time courses for WT and Kal7^KO ^mice showing the fEPSP slope in response to PP-LFS. LTD induction was at time zero (excluded from time course). C) Group data showing the effect of PP-LFS on fEPSP slope 60 min post-induction in WT and Kal7^KO ^animals (n = 8 animals, 2 slices/animal for each genotype). *, p < 0.05 compared to baseline.

**Figure 4:**
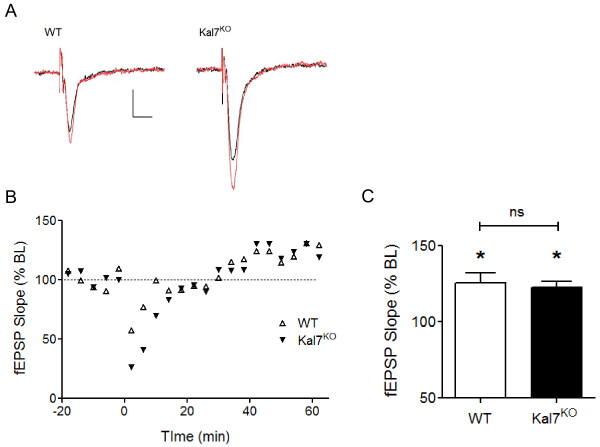
**NMDA receptor-independent LTP is unimpaired in Kal7^KO ^animals**. A) Example traces of evoked fEPSPS before (black) and 60 min after (red) LTP induction (200 Hz/2 sec in the presence of 3 μM CPP). Calibration bars: 0.1 mV, 20 ms. B) Representative examples from a WT and a Kal7^KO ^animal showing initial depression and slowly developing potentiation. LTP induction occurred at time zero. B) Group data showing the magnitude of NMDA receptor-independent LTP in WT and Kal7^KO ^animals 60 min post-induction (n = 7-8 animals, 2 slices/animal). *, p < 0.05 compared to baseline; ns, no significant difference between genotypes.

**Figure 5:**
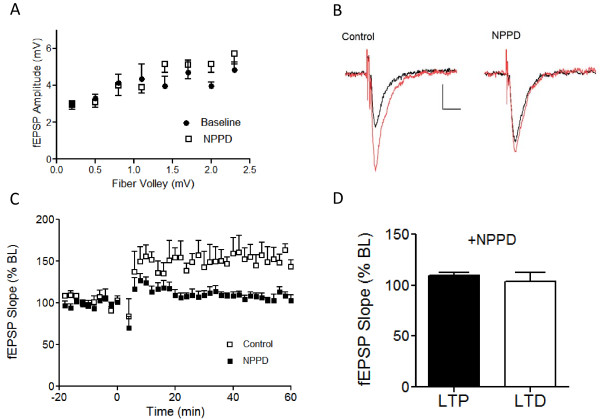
**Application of NPPD, an inhibitor of the Rho-GEF activity of Kal7, suppressed hippocampal LTP and LTD**. A) Input-output relationship for effects of NPPD (100 μM) on fEPSPs in 4 week old CD1 mice. B) Sample sweeps before (black) and 60 min after (red) LTP induction for control and NPPD-treated slices. The LTP induction protocol consisted of 3 trains of TBS. Calibration bars: 0.1 mV, 20 ms. C) Group time course for the effects of NPPD on LTP. D) Group data for the effect of NPPD on LTP (n = 5 slices/4 animals) or LTD (n = 7 slices/3 animals). The LTD induction protocol was the same as in Figure 3. For all experiments, NPPD (100 μM) was present for a 15 min pretreatment and throughout the experiment.

Briefly, C57BL/6 (WT or Kal7^KO^; used in Figures 1, 2, 3, 4) or WT CD1 mice (Figure 5) were decapitated under isoflurane anesthesia and the brains were harvested quickly and placed into ice-cold "cutting and incubating" (*CI*) solution composed of (in mM): 125 NaCl, 2.5 KCl,1.25 NaH_2_PO_4_, 25 NaHCO_3_, 0.5 CaCl_2_, 4 MgCl_2_, 4 MgSO_4_, 4 lactic acid, 2 pyruvic acid, 20 glucose, and 0.4 ascorbic acid, carboxygenated with 95% O_2 _- 5% CO_2 _(pH 7.3, 310 ± 5 mmol·kg^-1^). Transverse slices (350 μm) containing the hippocampus were cut using a vibratome (Microslicer, Dosaka EM, Kyoto, Japan). The slices were placed in a large incubating chamber containing *CI *solution at a temperature of 34 - 35°C for 30 minutes before being transferred to room temperature for at least 30 minutes prior to recording. Slices were then individually transferred to a recording chamber fixed to the stage of an Olympus BX50WI upright microscope. During recordings, slices were continuously perfused at 2 ml/min with artificial cerebrospinal fluid (aCSF) consisting of (in mM) 125 NaCl, 2.5 KCl, 1.25 NaH_2_PO_4_, 25 NaHCO_3_, 2 CaCl_2_, 2 MgCl_2_, and 15 glucose (pH 7.3, 310 ± 5 mmol·kg^-1^); pH was equilibrated by continuous bubbling with 95% O_2 _- 5% CO_2_.

### Electrophysiology

Hippocampal field excitatory postsynaptic potentials (fEPSPs) were recorded at room temperature from the stratum radiatum layer of CA1 using borosilicate glass microelectrodes (5-8 MΩ) filled with aCSF. A bipolar tungsten stimulating electrode (1 MΩ) was placed in the Schaffer collateral pathway approximately 250 μm lateral to the recording electrode. Extracellular fEPSPs were evoked at 0.05 Hz with an intensity that evoked half maximal responses. Slopes of fEPSPs were generated from linear regression of the rising phase (20-80% of the peak response) using pClamp9 (Molecular Devices, USA). Tests of statistical significance were based on Student's *t*-tests or ANOVA. Group data are reported as mean ± standard error of the mean (SEM).

### Knockout animal generation/breeding/genotyping

Kal7^KO ^mice were generated as previously described by breeding Kal7^KO/+ ^males and females which had been backcrossed into C57BL/6 more than 10 generations [17]; DNA prepared from ear-punches taken at weaning (P21) was used to genotype pups [17]. Littermate wildtype and Kal7^KO ^mice were used at ages P50-P70 for all extracellular field recordings, with the exception of the results shown in Figure 5, which used 4 week old CD1 mice.

## Results

### Kal7^KO ^mice exhibit normal AMPA receptor-mediated transmission

We characterized basal AMPA receptor-mediated transmission at CA3-CA1 synapses in the hippocampus by generating input-output curves using evoked field potentials (see example sweeps in Figure 1A). To address potential differences in the excitability of the Schaffer collateral axons, we first examined the relationship between stimulation intensity and presynaptic fiber volley amplitude. As shown in Figure 1B, there was no genotypic difference in axonal excitability. To address potential differences in synaptic strength, we next examined the relationship between presynaptic fiber volley amplitude and either fEPSP amplitude (Figure 1C) or slope (Figure 1D). There was no genotypic difference in basal synaptic strength of AMPA-mediated transmission.

### Kal7^KO ^mice exhibit deficits in NMDA receptor-dependent plasticity

In addition to regulating spine number, Kal7 interacts with other postsynaptic density proteins involved in synaptic function, and has been suggested to play a key role in activity-dependent plasticity [23-25]. Indeed, in single-cell LTP experiments in the Kal7^KO ^hippocampus we observed a pronounced decrease in potentiation [17]. In the next series of studies we characterized the role of Kal7 in hippocampal synaptic plasticity on a network level by performing hippocampal field recordings. Specifically, we examined LTP at Schaffer collateral-CA1 synapses in the hippocampus using trains of theta burst stimulation (TBS; each train contains 10 bursts delivered at 5 Hz, each burst consists of 5 stimuli at 100 Hz) as well as high frequency tetanic stimulation (HFS; 100 Hz/1 sec) in acute brain slices. As shown in the example sweeps in Figure 2A and group time courses in Figure 2B, WT mice, but not Kal7^KO ^littermates, showed marked potentiation following three trains of TBS. Group data in Figure 2C revealed that significant LTP could be induced in WT animals with either 1 or 3 TBS trains or with HFS, whereas Kal7^KO ^mice showed no LTP with 3 TBS or HFS, and a small but statistically significant LTP with 1 TBS that was significantly decreased compared to WT animals.

To confirm that the LTP produced by these paradigms was dependent on NMDA receptor activation, we performed the same stimulation paradigms in the presence of 3 μM of the NMDA receptor antagonist 3-(2-carboxypiperazin-4-propyl-1-phosphonic acid (CPP). Both TBS and HFS-induced LTP were dependent on NMDA receptor activation, as no significant LTP was induced using either paradigm when NMDA receptors were blocked (Figure 2D). We also examined the contribution of NR2B-containing receptors, as we have recently reported a deficit in NR2B-mediated signaling in Kal7^KO ^mice [22]. As shown in Figure 2D, blocking NR2B-containing receptors with ifenprodil (3 μM) completely prevented TBS-induced LTP in WT animals.

To determine whether the disruption of synaptic plasticity was specific to protocols that enhance synaptic transmission, we also examined the effects of Kal7 deletion on long term depression (LTD) at these same hippocampal synapses. Initial studies used a standard low frequency stimulation protocol (1 Hz/900 stimuli) commonly used for rat slice recordings, however we found no significant LTD in adult mice of either genotype using this protocol. Other studies have also reported that low frequency stimulation, which induces LTD in younger animals, is often ineffective in producing LTD in adult rats [26-29]. We therefore employed a modified induction protocol using paired-pulse low frequency stimulation (PP-LFS), which has previously been shown to be effective in inducing LTD in older rats [26,29,30]. As shown in the example traces in Figure 3A and group data in Figures 3B&3C, slices from WT mice showed significant LTD in response to PP-LFS (63 ± 9% of baseline, n = 8, p < 0.05). In contrast, slices from Kal7^KO ^mice showed no significant change after LTD induction (100 ± 10% of baseline, n = 8).

### Kal7^KO ^mice exhibit normal NMDA receptor-independent LTP

Because loss of Kal7 disrupted NMDA receptor-dependent LTP as well as LTD, we next examined whether this plasticity deficit extended to NMDA receptor-independent forms of plasticity at these same hippocampal CA3-CA1 synapses. To address this issue, we utilized a protocol that has been shown to induce a slowly developing and long-lasting form of potentiation that depends on calcium influx through voltage-gated calcium channels rather than through NMDA receptor channels [31]. In response to high frequency tetanic stimulation (200 Hz/2 sec) in the presence of the NMDA receptor antagonist CPP (3 μM), a transient depression was followed by a significant potentiation in WT animals measured 60 min after the induction protocol; individual sweeps and time courses are shown in Figures 4A &4B. Time courses across individual experiments were quite variable; nevertheless, as shown in the group data in Figure 4C, significant LTP was seen in both WT (125.7 ± 6.4% of baseline, n = 7 animals, p < 0.05) and Kal7^KO ^animals (122.6 ± 3.8% of baseline, n = 8 animals, p < 0.05) at 60 min post-induction. The amount of potentiation was not significantly different between genotypes. Thus, Kal7 is not required for this form of NMDA receptor-independent LTP.

### Kalirin GEF activity is essential for NMDA-dependent LTP and LTD

Kal7 is a large, multifunctional protein known to interact with multiple proteins localized to the PSD [15]; its ability to activate the Rho-GTPase Rac1 is essential for some, but not all of its effects [32]. To explore whether Kal7-Rac1 interactions are important for the expression of hippocampal LTP, we used 1-(3-nitrophenyl)-1H-pyrrol- 2,5-dione (NPPD), an inhibitor specific for the first GEF domain of Kalirin and Trio [33,34]. As shown in Figure 5A, NPPD (100 μM) had no significant effect on the input-output curve in WT mice. As shown in the example traces in Figure 5B and the group time courses in Figure 5C, in response to 3 trains of TBS, significant LTP was observed in control experiments (136.4 ± 7.4% of baseline, n = 5, p < 0.05), but not in NPPD-treated slices (109.5 ± 5.2% of baseline; n = 5), In addition to blocking LTP, NPPD also prevented LTD (PP-LFS paradigm) (Figure 5D). Since Kal7^KO ^mice failed to show LTP or LTD using these paradigms, these experiments were performed only on WT animals.

## Discussion

Previous studies of Kal7^KO ^mice revealed a decrease in CA1 hippocampal dendritic spine density, focal behavioral impairments in hippocampal-dependent tasks [17], and deficits in NMDA receptor-mediated signaling [22]. In the current studies, we examined hippocampal synaptic transmission and plasticity via a series of extracellular field potential recording experiments on the Kal7^KO ^mice and their WT littermates in order to better understand the network changes that have taken place in these animals and how they may contribute to the behavioral phenotypes.

Field potential input-output curves indicate that the Kal7^KO ^animals have normal axonal excitability and basal AMPA receptor-mediated transmission compared to WT littermates (Figure 1). We previously reported a decrease in sEPSP frequency and spine density in Kal7^KO ^mice compared to WT animals [17]. These changes could result from either a reduction in the number of synaptic contacts per axon, which would affect the input-output relationship, or a reduction in the number of axons, which would not affect the input-output curve, since it is normalized to the size of the presynaptic fiber volley. Our data suggest that there is a decrease in the number of axons in Kal7^KO ^animals. It is also possible that the fEPSP is not sensitive to relatively small changes in synapse density.

Given the changes seen in NMDA receptor function recently reported in Kal7^KO ^mice [22] and the difference in LTP at the single cell level [17], we next chose to examine NMDA receptor-dependent forms of plasticity at the network level. In Kal7^KO ^mice, hippocampal LTP in response to 1 or 3 trains of TBS at CA3-CA1 synapses was significantly impaired (Figure 2). Significant potentiation was also observed in WT animals using a HFS paradigm, which failed to induce LTP in Kal7^KO ^mice. Potentiation in response to either TBS or HFS was NMDA receptor-dependent, although stimulation in the theta frequency is a paradigm that is more relevant to the physiological range of synaptic activity [35]. We also found that low frequency stimulation-induced LTD, which requires activation of synaptic NMDA receptors [36], was likewise disrupted in Kal7^KO ^animals (Figure 3). In contrast, no genotypic deficit was seen in a non-NMDA receptor mediated form of LTP at these same synapses (Figure 4). These results suggest that Kal7 plays a critical and selective role in multiple forms of NMDA receptor-dependent synaptic plasticity.

We have previously reported that expression of the NR2B subunit of the NMDA receptor is decreased in PSDs purified from the hippocampus of Kal7^KO ^mice [17]. In addition, we found that signaling mediated by NR2B subunit-containing NMDA receptors is impaired in Kal7^KO ^animals, as shown by a decrease in the NMDA/AMPA ratio and a decreased sensitivity to the NR2B antagonist ifenprodil [22]. Thus, there may be a direct link between elimination of Kal7 and NR2B deficiency. The role of NR2B in the development of LTP and LTD has long been controversial [37-39]. However, recent studies have demonstrated that the presence of NR2B and its binding partners at the synapse is essential for induction of LTP in the hippocampus [40-42], and we found in the present studies that TBS-induced LTP required NR2B-containing receptors. Additionally, behavioral experiments have demonstrated that specific blockade of NR2B receptors prevents fear learning and conditioned place preference for drugs [43-45]. Interestingly, Kal7^KO ^mice exhibit specific deficits in both contextual fear conditioning and conditioned place preference for cocaine [17,19]. Future studies will explore the specific role of the NR2B subunit in the plasticity and behavioral deficits seen after Kal7 deletion.

Alternative splicing generates multiple Kalirin proteins from the *Kalrn *gene [46]. Mice engineered to lack exons encoding the first GEF domain of Kalirin (KalGEF1^KO^) lack Kal7 as well as the larger isoforms, Kal9 and Kal12 [20]. KalGEF1^KO ^mice show normal basal AMPA-mediated transmission in the hippocampus [21], as documented here for the Kal7^KO ^mice (Figure 1). Multiple trains of high frequency tetanic stimulation were used to analyze hippocampal LTP in WT and KalGEF1^KO ^mice, and a small decrease in potentiation was noted in the KO animals at 90 and 120 minutes after LTP induction [21]. However, this supra-physiological high frequency tetanic stimulation was the only induction paradigm attempted in the KalGEF1^KO ^mice. In the present study using Kal7^KO ^animals, we report a small but significant deficit in tetanus-induced LTP, and no deficit in a non-NMDA receptor mediated form of tetanus-induced LTP (calcium channel-dependent). This is in stark contrast to the profound deficits seen with TBS induction of LTP. As TBS more accurately reflects activity patterns observed *in vivo*, these results suggest that Kal7 plays an important role in physiologically-relevant plasticity paradigms.

Activation of Rac1 and actin remodeling are critical for the clustering of AMPA receptors and changes in dendritic spine morphology that occur following LTP induction [4,5,12,47]. However, there are numerous Rac1 GEFs at the PSD and any of these could be affecting these changes [14]. In order to examine more specifically the Rac GEF activity of Kal7, we chose to use NPPD, which inhibits the N-terminal GEF1 domain of Kalirin (the only GEF domain in Kal7) and its ortholog Trio, but does not affect the catalytic activity of other GEF proteins tested [34,48]. Previous studies from our lab have shown that NPPD is effective at inhibiting the first GEF domain of Kalirin in pituitary cells [33]. LTP and LTD induction were fully blocked in the presence of NPPD, while basal transmission was unaltered. Although there are other Rac GEFs at the PSD, Kal7 clearly plays an important and non-redundant role in activity-dependent synaptic plasticity. This may help to explain the learning related behavioral changes seen in Kal7^KO ^mice that are theoretically dependent on development of hippocampal LTP [17].

It is intriguing to speculate on the potential relationship between deficits in NMDA receptor-mediated synaptic plasticity with preservation of non-NMDA receptor-mediated forms of synaptic plasticity on the one hand, and the behavioral tests that are either impaired or preserved. Interestingly, Kal7^KO ^mice show deficits in behavioral tests related to fear and anxiety (elevated zero maze, passive avoidance), but perform normally on non-aversive tasks such as object recognition, radial arm maze and conditioned place preference for food [17,19]. Future studies will extend these results to explore the specific roles of Kal7 in other brain regions. For example, because Kal7 is also highly expressed in the amygdala, it will be interesting to explore the potential role of Kal7 in amygdala-dependent fear conditioning tasks, which have previously been shown to be impaired following deletion of Kal7 or Kalirin GEF1.

## Conclusions

Kal7 is an essential and selective modulator of NMDA receptor-dependent synaptic plasticity in the hippocampus.

## Authors' contributions

FL-C, DDK, ESL, BAE and REM contributed to conception and design of experiments. FL-C and LZ collected all data. FL-C, LZ and ESL performed all data analysis. All authors contributed to data interpretation. DDK and ESL wrote the original draft of the manuscript. All authors contributed to manuscript revision and approved the final manuscript.
